# Clinical features and prognostic factors of anti-MDA5 antibody-positive dermatomyositis-associated interstitial lung disease with spontaneous mediastinal emphysema: a retrospective case series of nine patients

**DOI:** 10.3389/fmed.2026.1804132

**Published:** 2026-04-10

**Authors:** Sulan Ge, Youyu Zhao, Fangfang Qu, Bailin Wu

**Affiliations:** 1 Department of Radiology, The Second Hospital of Hebei Medical University, Shijiazhuang, China; 2 Department of Respiratory and Critical Care Medicine, The Second Hospital of Hebei Medical University, Shijiazhuang, China

**Keywords:** anti-melanoma differentiation-associated gene 5 antibody, dermatomyositis, high-resolution computed tomography, interstitial lung disease, MDA5-CT score, serum ferritin, spontaneous pneumomediastinum

## Abstract

**Objective:**

Spontaneous pneumomediastinum (SPM) is a life-threatening complication of anti-melanoma differentiation-associated gene 5 antibody-positive dermatomyositis (MDA5 + DM) associated interstitial lung disease (ILD), particularly in patients with rapidly progressive ILD (RP-ILD), which carries an extremely high mortality rate. This study aimed to investigate the clinical characteristics, imaging features, and prognostic factors of MDA5 + DM-ILD patients with SPM to facilitate early detection and risk stratification of this high-risk population.

**Methods:**

The study retrospectively analyzed the clinical data of nine patients diagnosed with MDA5 + DM-ILD complicated by SPM at the center between June 2022 and February 2025. Collected data included demographic characteristics, laboratory biomarkers [including serum ferritin and lactate dehydrogenase (LDH)], high-resolution computed tomography (HRCT) findings assessed through the validated MDA5-specific computed tomography (MDA5-CT) scoring system, treatment regimens, and clinical outcomes.

**Results:**

The cohort included 4 male and 5 female patients, with a median age of 62 years (range 42–69 years). All patients presented with RP-ILD and developed SPM during the disease course, with a median hospital stay of 10 days (range 4–31 days). The overall mortality rate was 87.5% (7/8, excluding 1 patient lost to follow-up). All non-survivors had high-risk MDA5-CT scores (>18 points), accompanied by significantly elevated serum ferritin [median 1936 ng/mL, interquartile range (IQR) 1,119–3,204 ng/mL] and LDH (median 433 U/L, IQR 327–646 U/L). The only surviving patient had a low-risk MDA5-CT score (≤18 points).

**Conclusion:**

Patients with MDA5 + DM-ILD-SPM carry an extremely poor prognosis and high short-term mortality. An MDA5-CT score >18 is a strong predictor of mortality, particularly within the first 6 months after onset, with the highest risk in the first 3 months. Combining elevated serum ferritin, increased LDH, and tracheal dilation (>18 mm) may aid in early identification of high-risk patients for timely intervention.

## 1 Introduction

Anti-melanoma differentiation-associated gene 5 antibody-positive dermatomyositis (MDA5 + DM) is a unique subtype of idiopathic inflammatory myopathy (IIM). Clinically, MDA5 + DM can be categorized into three phenotypes: (1) cutaneous-only disease; (2) chronic interstitial lung disease (ILD)-predominant disease; and (3) rapidly progressive ILD (RP-ILD)-predominant disease. The RP-ILD phenotype is associated with an extremely poor prognosis, with a mortality rate of up to 80% within 6 months of onset (1). Spontaneous pneumomediastinum (SPM) is an uncommon but severe complication in patients with ILD. Previous studies have reported that approximately 10% of MDA5 + DM patients develop SPM during the disease course (2). The development of SPM is significantly associated with a higher risk of respiratory failure and mortality in patients with RP-ILD [hazard ratio (HR) = 2.829] (3), and reduces median survival (13.6 months in the SPM group vs. 77.7 months in the non-SPM group) (3). However, existing data on the clinical characteristics, prognostic biomarkers, and risk stratification tools for MDA5 + DM-ILD patients with SPM remain limited, and the predictive value of the MDA5-CT scoring system in this high-risk population has not been validated to date. Accordingly, this study aimed to summarize the clinical and imaging features of MDA5 + DM-ILD patients with SPM, evaluate the prognostic performance of the MDA5-CT scoring system in this cohort, and provide evidence for risk-stratified clinical management to facilitate early identification and intervention in high-risk patients.

## 2 Materials and methods

### 2.1 Inclusion and exclusion standards

This study was approved by the Ethics Committee of the Second Hospital of Hebei Medical University (ID: 2025-R212). All procedures were performed in accordance with the ethical standards of the Declaration of Helsinki. Informed consent was waived due to the retrospective design of the study. A retrospective cohort study was conducted at the Department of Respiratory and Critical Care Medicine, the Second Hospital of Hebei Medical University. Patients admitted between June 2022 and February 2025 with a diagnosis of MDA5 + DM. All patients were managed by a multi-disciplinary team (MDT) consisting of rheumatologists, pulmonologists, and radiologists.

The inclusion criteria were as follows: (1) met the 2018 European Neuromuscular Centre (ENMC) classification criteria for IIM, which are recognized and compared with other international standards (4). And the 2022 Chinese expert consensus on the diagnosis and treatment of ILD associated with IIM (5); (2) positive serum anti-MDA5 antibody; (3) high-resolution computed tomography (HRCT) consistent with ILD; and (4) SPM was confirmed during hospitalization. Exclusion criteria included: (1) SPM caused by trauma or iatrogenic factors (e.g., central line placement and barotrauma from mechanical ventilation), or other identified non-spontaneous causes (e.g., Valsalva maneuver); and (2) incomplete clinical data.

### 2.2 Data collection

The electronic medical record system was used to collect the patient’s demographic information, clinical manifestations, laboratory tests (including inflammatory indicators, auto-antibodies, lymphocyte counts, and infectious pathogens), imaging findings, treatment plans, and clinical prognoses.

### 2.3 Imaging evaluation

All patients underwent chest HRCT (GE Gemstone Spectral CT) with optimized scan parameters, including tube voltage set at 120 kV and tube current at 320 mA, slice thickness and slice spacing both 1 mm, and scanning with breath-holding in the inspiratory phase to ensure high-resolution imaging of the lung parenchyma and small airways. Two senior radiologists performed blinded, independent evaluations, reaching consensus through discussion when disagreements arose. Assessment included:

*ILD pattern classification*: the classification was categorized according to the International Multidisciplinary Classification Criteria as non-specific interstitial pneumonia (NSIP), organizing pneumonia (OP), NSIP+OP mixed pattern, diffuse alveolar damage (DAD), and usual interstitial pneumonia (UIP) (6).

*Tracheal measurement and SPM assessment*: Tracheal diameter was measured at the level of the manubrium and document the timing and extent of SPM.

*MDA5-CT scoring*: the scoring system proposed by Xu et al. (7), was used to evaluate the extent of ground-glass opacities (GGO) and solid lesion in all five lobes of both lungs, and to calculate the total score. The MDA5 CT score, calculated as Σ(5-lobe GGO scores) + 2 × Σ(5-lobe solid lesion scores), is a critical diagnostic tool for assessing the severity of MDA5-associated lung conditions. A score >18 indicates high-risk status for developing RP-ILD and death, whereas a score of ≤18 is classified as low-risk (7).

*Detailed rules*:

*Lobar division*: Right upper, middle, and lower lobes + left upper and lower lobes (5 lobes total), scored separately.

(1) Single-lobe scoring criteria:

**Table:** 

Area of involvement	GGO/solid lesion score
0%	0
≤5%	1
5–25%	2
25–49%	3
50–75%	4
≥75%	5

(2) Total score calculation:

$$\begin{array}{l} \mathrm{MDA5}\;\text{total score}=\Sigma (5-\text{lobe}\;\mathrm{GGO}\;\text{score})+2\\ \times \;\Sigma (5-\text{leaf solid lesion score}) \end{array}$$

## 3 The results

This study included nine patients (four men and five women), with ages ranging from 42 to 69 years. The patients primarily exhibited clinical features of dyspnea, oxygen desaturation, and SPM. Short-term imaging studies indicated the exacerbation of interstitial pneumonia. The nine patients’ characteristics are outlined below. Seven of the deceased patients exhibited an MDA5 CT score of >18 (high risk). The SPM development occurred during admission or during the course of treatment, accompanied by severe immune suppression (lymphopenia) and inflammation [elevated levels of serum ferritin, lactate dehydrogenase (LDH), and C-reactive protein (CRP)], despite the administration of aggressive immune suppression therapy [including hormones and intravenous immunoglobulin (IVI)]. Another patient improved and was discharged but was subsequently lost to follow-up. At the follow-up visit, new pneumomediastinum was detected, with prognosis unknown. The overall mortality was 87.5% (7/8), excluding one patient lost to follow-up. Mediastinal emphysema, particularly in the context of MDA5 + DM, is a severe complication that can serve as a significant indicator of poor prognosis.

### 3.1 General clinical features

This study included nine cases of MDA5 + DM-ILD-SPM. The baseline characteristics of these patients are shown in Table 1. The study population included four men and five women, with a median age of 62 years (range 42–69 years). All nine patients met the clinical diagnostic criteria for clinically amyopathic dermatomyositis (CADM) established by the ENMC in 2018, defined as the presence of characteristic dermatomyositis skin manifestations without objective muscle weakness.

**Table 1:** Patient baseline characteristics (*n* = 9).

Case	Gender/age	Time from onset of initial symptoms to hospital admission	Initial symptom	Skin manifestations	Anti-MDA5 antibody	Anti-Ro-52 antibody	Initial oxygenation index (PaO₂/FiO₂)
1*	Male/62	1 month	Cough, shortness of breath	Mechanic’s hands, Gottron’s rash	Positive	Positive	100
2*	Male/45	2 month	Cough, shortness of breath	Mechanic’s hands, Gottron’s rash	Positive	Strong positive	71
3*	Male/59	1 month	Shortness of breath	Mechanic’s hands; Gottron’s rash	Strong positive	Strong positive	167
4	Female/51	2 week	Shortness of breath	Mechanic’s hands; Gottron’s rash; V-neck sign; Shawl sign	Positive	Positive	≥300
5*	Female/42	2 month	Cough, shortness of breath	Mechanic’s hands, Gottron’s rash	Positive	Positive	198
6*	Female/69	3 week	Cough, shortness of breath	Mechanic’s hands, Gottron’s rash	Strong positive	Moderately positive	142
7*	Male/65	1 week	Shortness of breath	Mechanic’s hands, Gottron’s rash	Positive	Positive	112
8	Female/60	1 year	Cough	Mechanic’s hands; Heliotrope rash; V-neck sign; Shawl sign; Gottron’s papules	Strong positive	Positive	276
9*	Female/62	10 month	Cough, shortness of breath	Mechanic’s hands, Gottron’s rash	Positive	Negative	53

MDA5 + DM, anti-melanoma differentiation-associated gene 5 antibody-positive dermatomyositis; ILD, interstitial lung disease; SPM, spontaneous pneumomediastinum; PaO₂/FiO₂, ratio of arterial partial pressure of oxygen to fraction of inspired oxygen; CADM, clinically amyopathic dermatomyositis; ENMC, European Neuromuscular Centre.

*Indicates patients who died during the follow-up period.

The most common initial presenting symptoms were cough and shortness of breath (six cases). Mechanic’s hands and Gottron’s rash were the most common cutaneous lesion, documented in all nine patients. Other dermatomyositis-specific cutaneous manifestations included heliotrope rash, V-neck sign, and Shawl sign (two cases). None of the patients exhibited objective proximal muscle weakness during the clinical course.

### 3.2 Biomarkers

Key Biomarkers are shown in Table 2. The median serum ferritin in the fatality group (seven cases) was 1936 ng/mL, and the median lactate dehydrogenase (LDH) level was 433 U/L, both significantly elevated. All patients exhibited lymphopenia (median lowest value 520/μL). CRP was also elevated in the fatality group. Additionally, pulmonary co-infections were documented in four patients (Table 2), including *Candida albicans*, *Pneumocystis jirovecii* pneumonia (PJP), *Aspergillus* species, Epstein–Barr virus (EBV), coronavirus disease 2019 (COVID-19), and *Mycoplasma pneumoniae*. These infections may contribute to the deterioration of respiratory status and potentially influence SPM development.

**Table 2:** Peak key laboratory indicators.

Case	Ferritin value (ng/mL)	LDH value (IU/L)	Lymphocyte lowest value (/μL)	C-reactive protein peak (mg/L)	D-dimer peak (μg/mL)	Co-infecting pathogens
1*	—	721	227	40.1	0.28	*Candida* + Pneumocystis + EBV
2*	—	646	100	38.4	3.55	—
3*	1936	433	539	40.1	0.25	*Aspergillus* + Pneumocystis
4	856	297	1,100	6.2	0.30	COVID-19
5*	1936	327	598	16.7	1.57	—
6*	847	316	670	29.6	0.35	—
7*	—	351	520	38.5	0.46	—
8	276	218	2,570	4.27	—	Mycoplasma pneumonia
9*	3,627	527	800	11	0.35	—

Co-infecting pathogens were identified via sputum culture, bronchoalveolar lavage fluid next-generation sequencing (NGS), or serum pathogen PCR testing; EBV, Epstein–Barr virus.

*Indicates patients who died during the follow-up period.

### 3.3 Imaging features and MDA5-CT scores

The imaging patterns of all patients were classified as NSIP+OP (eight cases) or UIP (one case). The clinical significance and diagnostic relevance of the MDA5-CT scores are detailed in Table 3. All seven deceased patients scored ≥18 (classified as high-risk group), with a median total score of 27 [interquartile range (IQR) 24–30; full range 19–32]. Among the two patients scoring <18 (low-risk group), one remained alive at the final follow-up, while the other was lost to follow-up after the initial post-baseline assessment.

**Table 3:** Imaging characteristics and MDA5-CT scores.

Cases	Interstitial pattern	Time of mediastinal emphysema	GGO total score	Solid lesion total score	MDA5 total score	Risk stratification	Tracheal width (mm)	Pneumonia prevalence (%)
1*	NSIP+OP	Day 1	15	6	27	High risk	20.0	44.77
2*	NSIP+OP	Day 1	11	4	19	High risk	20.4	30.44
3*	NSIP+OP	Day 5	12	10	32	High risk	19.2	9.61
4	NSIP+OP	Day 1	11	2	13	Low risk	16.1	4.67
5*	NSIP+OP	Day 12	12	7	26	High risk	18.1	17.72
6*	NSIP+OP	Day 13	10	7	24	High risk	21.1	39.97
7*	UIP	Day 1				High risk	20.3	4.93
8	NSIP+OP	Day 30	18	5	28	High risk	21.8	9.53
9*	NSIP+OP	Day 1	13	4	17	Low risk	16.9	25.77

AI Quantitative Analysis (using InferViewer software).

NSIP, Non-specific interstitial pneumonia pattern; OP, Organizing pneumonia pattern; UIP, usual interstitial pneumonia; GGO, Ground-glass opacity; SPM, Spontaneous pneumomediastinum; HRCT, High-resolution computed tomography.

The 7th patient did not undergo CT examination in time.

*Indicates patients who died during the follow-up period.

In five patients, SPM was detected on the first day of admission. Among them, in one patient, SPM had been confirmed by CT at an outside hospital, and presented to our hospital due to worsening dyspnea. The remaining four patients were admitted due to dyspnea, and SPM was found on their initial imaging examination. None of the patients was admitted solely for SPM. However, the remaining four patients (Cases 3, 5, 6, and 8), SPM developed during hospitalization (on Day 5, 12, 13, and 30, respectively). Six patients (Cases 2, 3, 4, 5, 7, and 9) were complicated with subcutaneous emphysema, which presented as air accumulation in the subcutaneous tissue of the neck or chest wall on chest CT, consistent with the previously reported correlation between SPM and subcutaneous emphysema in patients with MDA5 + DM (8). Patients in the high-risk group exhibited markedly enlarged tracheal diameters (median 20.3 mm).

### 3.4 Treatment and prognosis

All patients underwent intravenous methylprednisolone (IVMP) therapy, with five cases combined with IVIG. The initial dosage range was 1.0 mg·kg^−1^·d^−1^ for critically ill patients. Among the five patients who received combination therapy with IVIG, the dosage was 0.4 g·kg^−1^·d^−1^, with 3–5 consecutive days constituting one treatment course. Respiratory support modalities included high-flow nasal cannula (HFNC) and invasive mechanical ventilation (IMV) (Table 4).

**Table 4:** Treatment and prognosis.

Case	Immunosuppressive regimen (methylprednisolone: 1.0 mg·kg^−1^·d^−1^, IVIG: 0.4 g·kg^−1^d^−1^)	Respirator support	ECMO	Infection management	Length of hospital stay (Days)	Outcome
1*	IVMP 74 mg/d; IVIG 29.6 g/d × 5 d (total: 148 g) Tacrolimus: 1 mg twice daily (oral)	IMV	None	Fluconazole, Ganciclovir	10	Death
2*	IVMP 65 mg/d; IVIG 26 g/d × 3 d (total: 78 g)	IMV	None	Pivaloxilin-tazobactam	4	Death
3*	IVMP 75 mg/d; IVIG 30 g/d × 4 d (total: 112 g)	HFNC	None	Trimethoprim sulfamethoxazole(TMP-SMX)	7	Death
4	IVMP 62 mg/d; Tacrolimus: 1 mg twice daily(oral); Mycophenolate Mofetil: 1 g twice daily (oral)	HFNC	None	Nirmatrelvir/Ritonavir	31	Survival
5*	IVMP 58 mg/d; IVIG 23.2 g/d × 5 d (total: 116 g)	HFNC	None	Ceftriaxone/Tazobactam	15	Death
6*	IVMP 60 mg/d; Tacrolimus: 1 mg twice daily (oral); Tofacitinib: 5 mg twice daily (oral)	HFNC	None	None	13	Death
7*	Nintedanib 150 mg twice daily (oral)	HFNC	None	Meropenem	15	Death
8	IVMP 63 mg/d	HFNC	None	Levofloxacin	7	Missing
9*	IVMP 61 mg/d; IVIG 24.4 g/d × 3 d (total: 73.2 g); Nintedanib 150 mg twice daily (oral)	HFNC	None	None	8	Death

IVMP, Intravenous methylprednisolone; IVIG, Intravenous immunoglobulin; ECMO, Extracorporeal membrane oxygenation; TMP-SMX, Trimethoprim-sulfamethoxazole; IMV, Invasive mechanical ventilation; HFNC, High-flow nasal cannula.

Immunosuppressive regimens documented in this table are the core in-hospital treatment regimens for each patient; respiratory support refers to the highest level of respiratory support received by the patient during hospitalization.

Outcome definition: Survival was defined as survival for more than 24 months after the initial treatment.

*Indicates patients who died during the follow-up period.

Positive pressure ventilation (PPV) was administered to only two patients (Cases 2 and 3). Case 2 underwent endotracheal intubation immediately upon admission due to severe hypoxemia; however, SPM was already diagnosed on admission chest imaging (Day 1), before PPV. Therefore, SPM in this patient was spontaneous. Case 3 developed SPM on Day 5 of hospitalization; PPV (endotracheal intubation) was initiated later on Day 7 as a rescue measure for worsening respiratory failure. The other seven patients were managed with HFNC.

During hospitalization, seven patients died, with a median survival of 10 days, indicating that 50% of the patients survived less than 10 days, with a range of 4–15 days. A low-risk patient (Case 4) has survived for over 24 months following treatment with a combination of glucocorticoids and tacrolimus, demonstrating the potential of this therapy to improve outcomes in such patients.

Case 8 exhibited improvement and was discharged. However, the patient was subsequently lost to long-term follow-up. A follow-up examination before the patient was lost to follow-up revealed new mediastinal emphysema, and thus the final prognosis remains unknown.

### 3.5 Typical cases

#### 3.5.1 Typical case 1

A 62-year-old male patient presented with progressive respiratory symptoms including chronic intermittent cough (4 weeks), productive sputum, chest tightness, dyspnea, and low-grade fever, with significant clinical deterioration during the 7 days preceding admission. The patient had previously visited multiple hospitals where he was diagnosed with ILD and SPM. Treated with IVMP for inflammation, and cefoperazone sodium and sulbactam for infection, with poor therapeutic efficacy. Chest CT at our hospital revealed multiple patchy and linear areas of increased density in both lungs, most prominent near the pleural surfaces, which may be indicative of interstitial pneumonia or other inflammatory conditions. Physical examination included alertness, wheezing appearance, cyanosis of lips, coarse breath sounds bilaterally, and audible moist rales.

##### 3.5.1.1 Admission arterial blood gas

pH: 7.46; PaO₂: 60.8 mmHg; PaCO₂: 36.7 mmHg; FiO₂: 61%.

##### 3.5.1.2 Biomarkers

Anti-MDA5 antibody positive (+); anti-Ro-52 antibody: 79.10 AU/mL; and elevated anti-SS-A antibody: 48.70 AU/mL. Total lymphocytes: 207/μL; CD4-positive T lymphocyte (CD4 + T cell): 29/μL. Inflammatory markers: CRP: 40.10 mg/L. LDH: 721.0 U/L and creatine kinase myocardial band (CK-MB): 85.0 U/L. Liver function: albumin: 28.6 g/L and alanine aminotransferase (ALT): 60.0 U/L. Sputum culture: *Candida albicans* (3+). Viral infection: Cytomegalovirus (CMV) DNA: 1.93 × 10^3^ copies/mL and EBV DNA: 7.07 × 10^2^ copies/mL. Complete blood count: white blood cell count: 13.24 × 10^9^/L and neutrophil percentage: 88.67%.

##### 3.5.1.3 Treatment course

**Figure 1:**
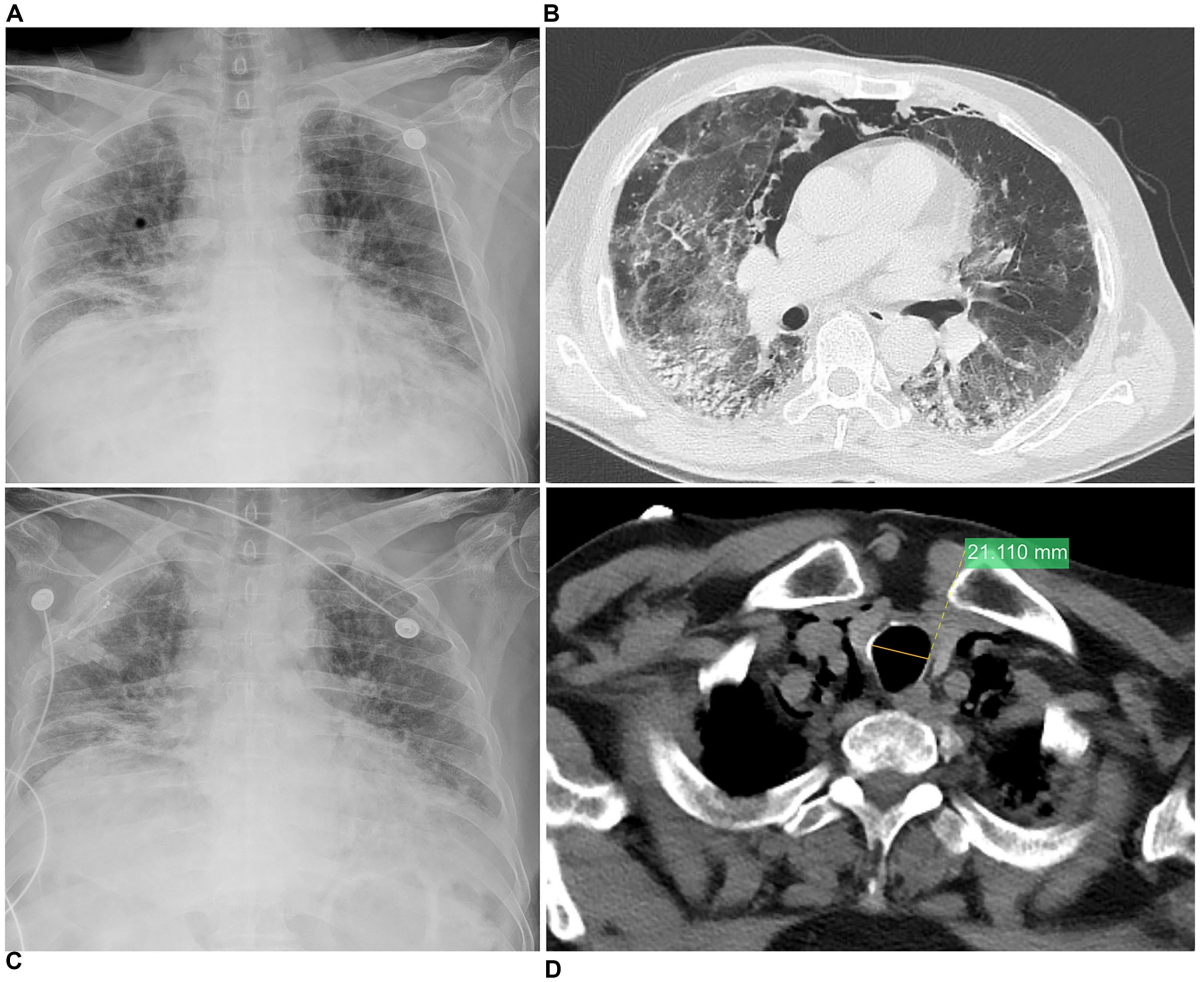
Chest imaging of case 1 (62-year-old male patient, corresponds to typical case 1). **(A)** Admission chest X-ray showing multiple GGO and patchy infiltrates in both lungs. **(B)** Day 3, lung window CT showed bilateral GGO, patchy infiltrates, reticular changes, and traction bronchiectasis, most prominent in the lower lobes of both lungs, with SPM. **(C)** Mediastinal window shows slight tracheal dilation. **(D)** On the fifth day, bedside chest X-ray showed that multiple areas of GGO and patchy exudation in both lungs, reduction in the extent of right lower lobe linear opacities and left lower field lesions compared to previous imaging.

Upon admission, chest X-ray showed multiple areas of GGO and patchy infiltrates in both lungs (Figure 1A). A comprehensive treatment regimen was initiated, encompassing meropenem for the control of bacterial infections, and fluconazole for antifungal therapy. Nasal high-flow oxygen therapy was used to support respiratory function. Immunosuppressive therapy was administered, involving IVMP (40 mg twice daily, approximately 1.0 mg·kg^−1^·d^−1^) and IVIG (25 g daily for 5 days, approximately 0.4 g·kg^−1^·d^−1^) to modulate the immune response. On the sixth day of hospitalization, the patient developed coughing, with marked exertional dyspnea and decreased oxygen saturation. The chest CT revealed mediastinal emphysema and significantly enlarged bilateral GGO compared to previous imaging (Figures 1B,C). Oral tacrolimus 1 mg twice daily was initiated. On the 10th day of hospitalization, the patient developed somnolence and decreased oxygen saturation. Oxygen concentration was increased to 90% with pulse oximetry fluctuating between 89 and 91%. A subsequent bedside chest X-ray (Figure 1D) revealed multiple areas of GGO and patchy infiltrates in both lungs. The patient refused admission to the intensive care unit (ICU), declined further endotracheal intubation, and invasive mechanical ventilation. The patient subsequently died (Figure 1).

#### 3.5.2 Typical case 2

**Figure 2:**
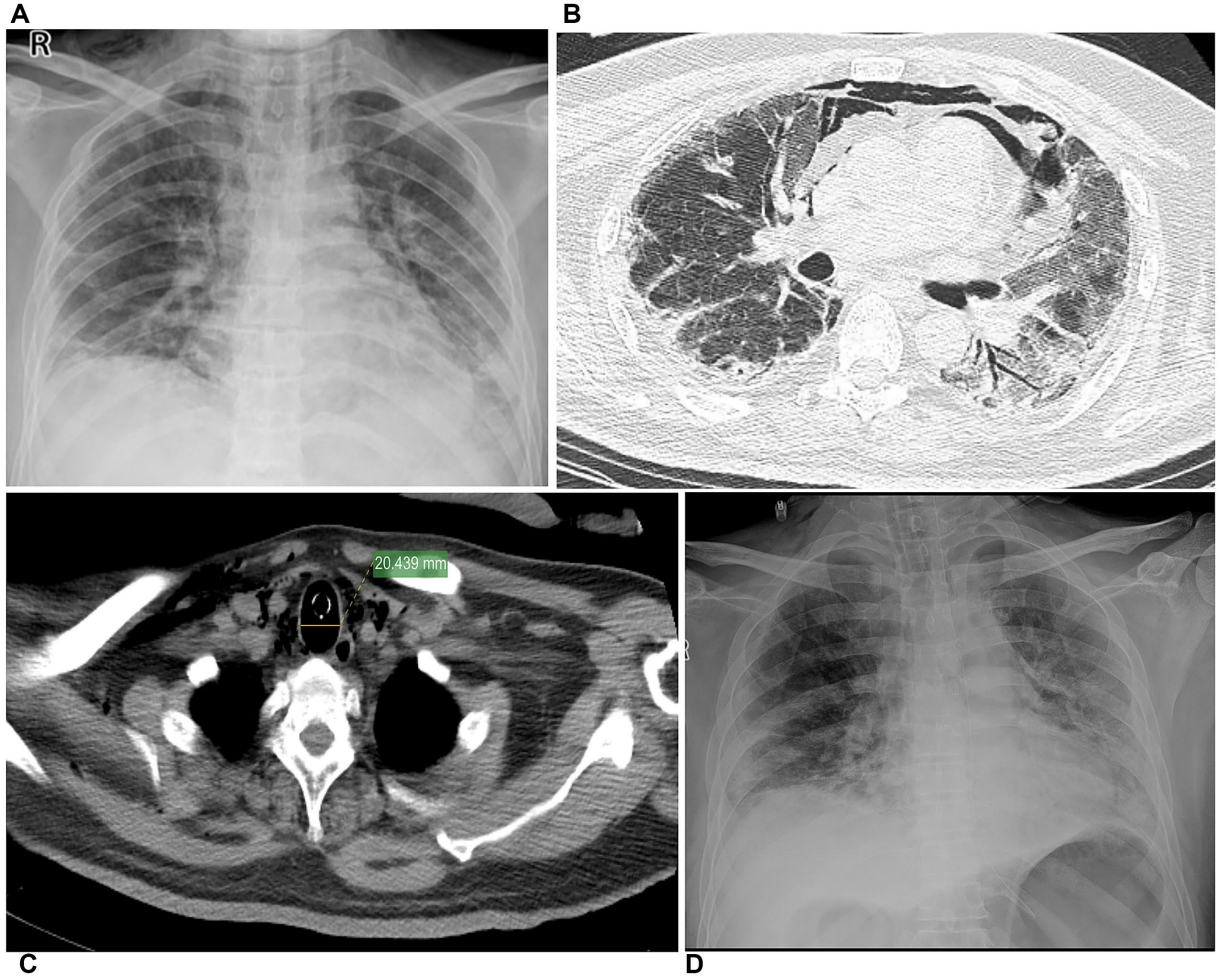
Chest imaging of case 2 (45-year-old male patient, corresponds to typical case 2). **(A)** Chest X-ray on admission, multiple GGO, and patchy infiltrates, more prominent in the left lung, and minimal air was observed in mediastinum. **(B)** The lung window revealed multiple air pockets in the mediastinal space and cervical roots, while the lung window showed multiple patchy, mottled, and GGO predominantly in the peripheral regions of both lungs. **(C)** Mediastinal window showing endotracheal tube and tracheal dilation. **(D)** On the second day post-admission, X-ray revealed multiple exudative lesions in both lungs, with a slightly more extensive distribution compared to previous imaging. Minimal air was observed in both cervical roots and mediastinum.

A 45-year-old male patient was admitted with a two-month history of intermittent cough and expectoration, which had significantly worsened over the past 5 days. The symptoms initially manifested as cough with white sputum production. About 45 days before admission, the patient developed additional symptoms including hoarseness, dyspnea on exertion, and fatigue. One month prior to admission, he was diagnosed with dermatomyositis-associated interstitial pneumonia and treated with methotrexate, cyclosporine, ganciclovir, and trimethoprim–sulfamethoxazole combination therapy, showing clinical improvement before discharge. However, his respiratory symptoms acutely worsened 5 days before presentation, with worsening cough and dyspnea accompanied by oxygen saturation levels of 80–90%. Upon admission in the emergency department, arterial blood gas analysis revealed severe hypoxemia (PaO₂: 35.4 mmHg) and hypocapnia (PaCO₂: 25.9 mmHg). Despite immediate oxygen supplementation, his respiratory status continued to deteriorate. Subsequently, endotracheal intubation and mechanical ventilation were performed. Initial chest radiography (Figure 2A) showed bilateral GGO and patchy infiltrates, more prominent in the left lung, and minimal air was observed in mediastinum. Subsequent thoracic computed tomography (Figures 2B,C) confirmed extensive bilateral GGO with left-sided predominance, along with pneumomediastinum and bilateral cervical subcutaneous emphysema. Physical examination revealed bilateral coarse breath sounds with prominent wet rales.

##### 3.5.2.1 Biomarkers

Myositis antibody panel: anti-MDA5 positive; immunological markers: Immunoglobulin G: 20.9 g/L, inflammatory markers: CRP: 38.40 mg/L; erythrocyte sedimentation rate (ESR): 22 mm/h; LDH: 646 U/L; and D-dimer: 3.55 mg/L. The sputum culture was negative.

##### 3.5.2.2 Treatment course

Upon admission, the patient received moxifloxacin for anti-infective therapy, IVMP 80 mg daily for anti-inflammatory treatment, and sulfamethoxazole for Pneumocystis pneumonia prophylaxis. On the second day of admission, the patient’s condition deteriorated critically. A repeat bedside chest X-ray (Figure 2D) showed multiple exudative lesions in both lungs, showing slight progression compared to previous findings. Minimal air trapping at bilateral cervical roots. IMV was initiated. To modulate the immune response, a 25 g IVIG was administered. On the fourth day of hospitalization, the patient’s condition continued to deteriorate with oxygen saturation dropping to approximately 50%. Despite sustained mechanical ventilation, the patient’s oxygenation failed to improve. Treatment proved ineffective, and the patient died (Figure 2).

## 4 Discussion

This study of nine patients with MDA5 + DM-ILD-SPM demonstrates that this clinical phenotype is characterized by rapid progression, refractory hypoxemia, and extremely high short-term mortality. The MDA5-CT score (>18 points) was 100% predictive of mortality in this cohort, and tracheal dilation (>18 mm) may serve as an additional imaging warning sign. These findings provide a practical risk stratification approach for early identification and intervention.

### 4.1 Imaging assessment: quantitative scoring and early warning signs

Imaging assessment and quantitative scoring are critical for risk stratification. Traditional ILD severity assessment tools (e.g., Warrick score) lack specificity for the MDA5 + DM subtype. Therefore, Xu et al. (7) developed an MDA5-specific CT scoring system in 2021. Quantifying the extent of GGO and solid changes, the system provides a relatively accurate and objective basis for prognostic assessment. In this study, all seven deceased patients had an MDA5-CT score >18 points (high risk), while one out of the two patients with a score ≤18 points achieved long-term survival. This is the first confirmation in the high-risk subgroup complicated by SPM that an MDA5-CT score >18 points serves as a potential early warning signal for mortality.

The pathological mechanism of SPM in MDA5 + DM patients is closely associated with CT features. The essence of the mechanism lies in the “disruption of alveolar-interstitial mechanical integrity” based on severe interstitial lung lesions. The classic “Macklin effect” can explain the imaging evolution in this group of patients (8). This phenomenon refers to alveolar rupture caused by increased intrapulmonary pressure or fragile alveolar walls, allowing air to enter the mediastinum along the bronchovascular sheath. In the patients of this study, the underlying disease led to DAD, and eight out of the nine cases presented with a NSIP+OP mixed pattern—an imaging finding that Chen et al. (9) confirmed as an independent predictor of RP-ILD. Pathologically, this pattern corresponds to the coexistence of alveolar septal inflammation (NSIP) and intra-alveolar organization (OP), resulting in decreased compliance, increased fragility of lung tissue, and uneven mechanical stress within the lung parenchyma. External factors such as severe coughing or PPV in patients only serve as precipitating triggers, further disrupting the mechanical balance of lung tissue and making specific areas (mediastinum, neck, and subcutaneous tissue) prone to tissue tearing. This mechanism is also supported by recent studies: Bond et al. (10) reported a case of MDA5 + DM dermatomyositis in which pneumothorax, pneumomediastinum, and pneumopericardium developed within a short period, and autopsy confirmed severe pulmonary inflammation and structural destruction.

Secondly, tracheal dilation is an indirect marker of reduced lung elasticity. The median tracheal diameter in the high-risk group was 20.3 mm, which is significantly higher than the reference value for healthy adults (15.2 ± 1.9 mm) (11). Although this sign has rarely been reported in previous literature, the pathophysiological significance is worthy of in-depth consideration. Severe pulmonary interstitial fibrosis leads to decreased elastic recoil of the lungs and increased radial traction, resulting in passive tracheal dilation. Therefore, tracheal dilation can serve as a marker of severe of “pulmonary sclerosis,” indicating that the lung tissue has lost normal constraints on airway morphology. Under these conditions, any factor causing fluctuations in intrathoracic pressure is more likely to induce barotrauma.

### 4.2 Serum biomarkers: reflecting inflammatory storm and tissue injury

Serum biomarkers reflect intense inflammation and tissue damage. In addition to imaging features, serum biomarkers also provide important evidence for prognostic assessment. In this study, patients with fatal outcomes exhibited markedly elevated ferritin (>1,000 μg/L) and LDH > 400 IU/L—both independently predicting RP-ILD progression and mortality (12, 13). Elevated ferritin indicates uncontrolled systemic inflammation, while increased LDH reflects extensive tissue damage involving multiple organs such as the lungs, liver, and myocardium. Notably, the LDH levels of all deceased patients exceeded the risk threshold for RP-ILD (>300 IU/L, odds ratio OR = 3.189) (12).

Meanwhile, hypoalbuminemia, electrolyte disturbances, and severe lymphopenia were prevalent among the patients, which reflect the systemic wasting, malnutrition, and immune dysfunction associated with RP-ILD (14, 15). These abnormalities, superimposed on high-dose immunosuppressive therapy, significantly increase the risk of opportunistic infections (such as PJP and *Aspergillus* infections observed in this cohort). Therefore, dynamic monitoring of ferritin, LDH, organ function, and nutritional and metabolic indicators is crucial for assessing disease activity and adjusting treatment regimens.

Anti-Ro-52 antibodies frequently coexist with anti-MDA5 antibodies, and previous studies have shown that the antibodies are associated with more severe ILD and poor prognosis (16). In this cohort, eight patients tested positive. Notably, all seven patients who died during follow-up were anti-Ro-52 antibody-positive, and they presented with higher baseline disease severity, as evidenced by initial oxygenation index consistently <200. In contrast, the only anti-Ro-52 antibody-negative patient (Case 9) also died, indicating that anti-Ro-52 antibody positivity is not an exclusive predictor of mortality but may potentiate disease severity in the context of anti-MDA5 antibody positivity. These findings suggest a potential link between anti-Ro-52 antibody positivity and a more severe clinical phenotype in patients with MDA5 + DM-ILD-SPM. However, due to the small sample size of this single-center retrospective study, this observed association lacks statistical power and requires further validation in large-scale prospective cohorts.

### 4.3 Clinical management implications: risk-based stratified management

The findings of this study have direct implications for clinical management. Despite intensive conventional regimens combining glucocorticoids with IVIG and/or calcineurin inhibitors, mortality remains alarmingly high among this high-risk patient cohort. These findings suggest that intensified and individualized treatment is required for patients with an MDA5-CT score >18 complicated by SPM.

For critically ill patients, early initiation of multi-targeted immunosuppressive therapy should be considered. Recommended regimens include triple therapy combining glucocorticoids with tacrolimus (or cyclosporine) and cyclophosphamide (17–19), or alternatively, Janus kinase (JAK) inhibitors (e.g., tofacitinib) as first-line agents (20, 21). A multidisciplinary approach involving rheumatology, pulmonology, critical care, and radiology teams is essential for optimal outcomes. Respiratory support requires judicious use of non-invasive ventilation to avoid worsening mediastinal emphysema (22). A refractory respiratory failure may necessitate evaluation for extracorporeal membrane oxygenation (ECMO) or therapeutic plasma exchange (TPE) (23). For patients with lower scores (≤18 points), treatment based on corticosteroids combined with calcineurin inhibitors may be considered (24, 25), but close follow-up is essential to monitor for disease progression.

Regardless of risk stratification, infection prevention and management should be prioritized in immunosuppressed patients. Due to severe CD4 + T lymphocyte depletion and immunosuppressive therapy, these patients have a significantly increased risk of PJP infection. From an imaging perspective, PJP mainly causes inflammatory exudation in the alveolar spaces, with typical CT findings of diffuse butterfly-like GGO. Anti-infective therapy had limited effect on improving prognosis. This suggests that underlying disease activity is the fundamental driver of poor outcomes. In addition, active correction of systemic complications such as hypoalbuminemia and electrolyte disturbances is necessary, along with dynamic monitoring of coagulation function—notably, significantly elevated D-dimer levels observed in some patients in this cohort suggest hypercoagulability and microthrombosis risk. Clinicians should maintain a high level of alertness for thromboembolic events, particularly pulmonary embolism (14), to improve overall patient outcomes through comprehensive management.

### 4.4 Study limitations and future directions

This study has several limitations that should be acknowledged. This single-center, retrospective case-series analysis, while providing valuable insights, is limited by its small sample size and the loss of one patient to follow-up, which may introduce bias and restrict the generalizability of the findings. Further validation of the MDA5-CT score’s predictive value in SPM patients requires future studies with larger cohorts and prospective designs, consistent with the development of similar clinical models in MDA5 dermatomyositis. Future studies should also explore whether new warning signs, such as air trapping, can be quantified and incorporated into a comprehensive scoring model. Additional clinical evidence is required to determine the most effective immunotherapy protocols and the appropriate timing for ECMO intervention in this high-risk population.

## 5 Conclusion

This study confirms that patients with MDA5 + DM-ILD-SPM have an extremely poor prognosis and high short-term mortality. For the first time, this study validated the predictive value of an MDA5-CT score > 18 points for short-term mortality in this high-risk SPM subgroup. The study also found that tracheal dilation (>18 mm) can serve as an imaging marker of decreased lung elasticity and a potential early warning sign for SPM. Combining the MDA5-CT score with elevated serum ferritin and LDH levels enables a more comprehensive early warning system to identify the highest-risk patients. These findings also support that severe underlying disease activity is the primary driver of SPM in most patients, although PPV should still be used cautiously in these vulnerable lungs. The potential association between anti-Ro-52 antibody positivity and adverse outcomes warrants further investigation. For high-risk patients, early multidisciplinary collaboration and aggressive multi-targeted immunosuppressive therapy should be considered, with close monitoring for infections and thromboembolic events. However, due to the small sample size and single-center retrospective design, the prognostic value of these factors requires validation in larger, prospective studies.

## Data Availability

The original contributions presented in the study are included in the article/supplementary material, further inquiries can be directed to the corresponding authors.
